# Role of Natural Killer Cells in HIV-Associated Malignancies

**DOI:** 10.3389/fimmu.2017.00315

**Published:** 2017-03-21

**Authors:** Fabio E. Leal, Thomas A. Premeaux, Mohamed Abdel-Mohsen, Lishomwa C. Ndhlovu

**Affiliations:** ^1^Programa de Oncovirologia, Instituto Nacional de Cancer, Rio de Janeiro, Brazil; ^2^Department of Tropical Medicine, Hawaii Center for AIDS, John A. Burns School of Medicine, University of Hawaii, Honolulu, HI, USA; ^3^Blood Systems Research Institute, San Francisco, CA, USA; ^4^University of California, San Francisco, CA, USA

**Keywords:** HIV, cancer, natural killer cells, ADCC, antiretroviral therapy, lymphoma, eradication and control

## Abstract

Now in its fourth decade, the burden of HIV disease still persists, despite significant milestone achievements in HIV prevention, diagnosis, treatment, care, and support. Even with long-term use of currently available antiretroviral therapies (ARTs), eradication of HIV remains elusive and now poses a unique set of challenges for the HIV-infected individual. The occurrence of HIV-associated non-AIDS-related comorbidities outside the scope of AIDS-defining illnesses, in particular non-AIDS-defining cancers, is much greater than the age-matched uninfected population. The underlying mechanism is now recognized in part to be related to the immune dysregulated and inflammatory status characteristic of HIV infection that persists despite ART. Natural killer (NK) cells are multifunctional effector immune cells that play a critical role in shaping the innate immune responses to viral infections and cancer. NK cells can modulate the adaptive immune response *via* their role in dendritic cell (DC) maturation, removal of immature tolerogenic DCs, and their ability to produce immunoregulatory cytokines. NK cells are therefore poised as attractive therapeutic targets that can be harnessed to control or clear both HIV and HIV-associated malignancies. To date, features of the tumor microenvironment and the evolution of NK-cell function among individuals with HIV-related malignancies remain unclear and may be distinct from malignancies observed in uninfected persons. This review intends to uncouple anti-HIV and antitumor NK-cell features that can be manipulated to halt the evolution of HIV disease and HIV-associated malignancies and serve as potential preventative and curative immunotherapeutic options.

## Introduction

Since the introduction of antiretroviral therapy (ART), life expectancy of people living with HIV (PLHIV) has notably improved, and the gap between the uninfected population ranging from 60 to 90%, of normal life expectancy is narrowing in regions of the world among those with access to ART (1). Before the availability of ART, immune suppression-related complications represented the predominant cause of mortality among HIV-infected individuals. Incidence rates of non-Hodgkin lymphoma (NHL) and Kaposi sarcoma (KS) were more than 100 times higher in the pre-ART era and were classified, together with cervical cancer, as AIDS-defining cancers (ADCs) (2). Overtime, as ART became the standard of care, prolonged use has lead to a remarkable improvement in immune status, dramatically reducing ADC rates (ratio of ART to no-ART) by 0.61 per year (3).

In the United States, despite the sharp decline in the incidence of ADCs, increased risk of developing specific types of NHL such as Burkitt lymphoma and classical Hodgkin lymphoma (HL) has evolved (4, 5). A meta-analysis of standardized incidence ratios from 18 studies showed that infection-related cancers such as anal, liver and HL, as well as smoking-related cancers such as lung, kidney, and oral cancers, had an increased incidence among the HIV population despite being on ART, with lymphomas being the most frequent type of cancer observed (6). A separate comprehensive epidemiological study showed that the PLHIV also had significantly higher rates of these now termed “non-AIDS defining cancers” (NADCs) when compared to matched HIV-negative individuals, suggesting the likelihood of other etiologies besides an increase in life expectancy as predisposing the HIV population to develop these types of cancers more frequently (7). In fact, NADCs have now become one of the leading causes of mortality among HIV-infected individuals (8, 9). Surprisingly, however, the incidences of breast and prostate cancers have significantly declined in HIV-infected persons, suggesting that HIV infection, ART, or other viral–host interactions have differential impacts on cancer risk in this population (10, 11). The increased incidence of NADC despite viral suppression and CD4 T-cell recovery in the era of ART raises important key mechanistic questions in the oncogenesis of NADC. The direct oncogenic effect of HIV, HIV-induced immunodeficiency, and chronic inflammation, as well as ART toxicities are some of the plausible mechanisms that are being investigated (12). Complete immune recovery after prolonged ART is variable and underscores that harnessing specific components of the host immune response may play a vital role in preventing NADC.

Chronic immune activation and immune senescence contribute to immune dysfunction in chronic HIV infection and partially persist even after CD4 T-cell count recovery and viral suppression by ART (13). Such processes lead to immune exhaustion/senescence, thereby facilitating reactivation of other latent viral infections, such as Epstein–Barr virus (EBV). Besides HIV, all ADCs and the majority of NADCs appear to be associated with several chronic viral infections (12), justifying a new way to categorize HIV malignancies into infection-related and non-infectious-related cancers. KS is intimately associated with HHV-8 infection (14), and cervical cancer is almost always caused by HPV infection (15). B-cell lymphoproliferative disorders are frequently associated with EBV infection, and such association is even more common in HIV-infected individuals, ranging from 60 to 100% (5, 16). Viral co-infections are present in NADC. The incidence of hepatocellular carcinoma has progressively increased among HIV-infected persons in the last decade (17). Merkel cell carcinoma, recently reported to be associated with Merkel cell virus, has a 20-fold increased risk among HIV-infected individuals (18). The potential direct effects of HIV in modulating oncogenes are under investigation, but how HIV impacts the oncogenic potential of other chronic viral infections is unclear (19–21). Persistent immune alterations may play a critical role in the oncogenic process in this population and deserve special attention, particularly in the context of co-infections.

## Natural Killer (NK) Cells: A Critical Immune Player in Antitumor and Anti-HIV Immunity

Since the discovery of NK cells 40 years ago, a plethora of research has uncovered their phenotypic and functional capacity against virally infected and tumor cells (22–25). NK cells are CD3− multifunctional effector lymphocytes defined based on levels of CD56 and CD16 expression (26), the majority (>90% of NK cells) in the peripheral blood being CD56dim and predominantly cytotoxic upon activation, thereby releasing pro-apoptotic cytoplasmic granules composed of granzymes and perforins. CD56dim NK cells can also induce cytolysis *via* induction of Fas/FasL-dependent or TRAIL-dependent apoptotic signals. In addition, a minority of NK cells express the FcγRIIIA receptor (CD16) that binds to the constant (Fc) domain of IgG antibodies that can bind to viral antigens expressed on the surface of infected cells. This antibody conjugation of NK-cell and antibody-coated target cell, strongly mediating NK-cell activation, is known as antibody-dependent cell-mediated cytotoxicity (ADCC) (27). A distinct subset of CD56bright cytokine-producing NK cells with a limited cytotoxic capacity is more abundantly present in lymph nodes (28). By producing IFN-γ, TNF-α, IL-10, and chemokines, this NK subset predominantly modulates other subsets of lymphocytes, thereby regulating dendritic cell maturation, differentiation of helper T cells, and B- and T-cell-specific immune responses (29, 30).

To understand the NK-cell effector functions, it is paramount to take into consideration the balance between activating and inhibitory signals (31) that drive NK-cell cytotoxicity. NK-cell activation relies on stimulatory signals capable of overcoming the steady inhibitory state that is maintained by signaling through inhibitory receptors. Self-recognition of MHC-I proteins through C-type lectin receptor NKG2A and inhibitory killer cell immunoglobulin-like receptors (KIRs) represent the physiological interaction between NK and target cells. The absence of recognition of “self” by inhibitory receptors characterizes the “missing-self” phenomenon and lowers the activating threshold. NK cells become more susceptible to activation, especially if activating molecules are expressed in infected or transformed target cells and recognized by activating receptors, characterizing the altered-self phenomenon. Activating C-type lectin receptor NKG2D recognizes the altered self-state of infected or transformed cells and triggers NK-cell cytolytic activity. Other surface molecules, such as natural cytotoxic receptors Nkp30, Nkp44, and Nkp46, and activating KIRs also contribute to NK-cell activation process and are critical to determine whether NK cells will be activated to target infected or transformed cells (27, 31).

Both HIV infection and oncogenesis lead to a downregulation of surface MHC-I expression as a way to avoid T-cell recognition but in turn renders target cells more susceptible to NK-cell-mediated cytolysis. However, HIV has developed immune evasion mechanisms *via* the viral protein Nef, thereby leading to preferential downregulation of HLA-A and -B, and preserving expression of HLA-C and -E (32). Therefore, HIV prevents NK activation as well as CTL recognition of infected cells. Besides interfering with self-recognition, HIV infection and cancer can induce expression of stress signaling molecules, in particular MHC class I polypeptide-related sequence A/B (MICA/MICB). More importantly, HIV leads to persistent activation and consequently T cell and NK-cell immune exhaustion. Despite viral suppression and normal CD4 T-cell counts in the majority of HIV-infected persons on ART, NK-cell phenotype and functionality are not fully restored, suggesting that these individuals may be more susceptible to long-term comorbidities associated with immune dysfunction, such as HIV-related malignancies (33).

## The Interplay between the Tumor Microenvironment and NK-Cell Immunity

The process by which the immune system can promote or suppress tumor growth and development is based on animal models and data from cancer patients and has evolved to define the concept of cancer immunoediting (34). Tumor immunoediting is comprised of three phases: elimination, equilibrium, and escape. The elimination phase is when immune cells target cancer cells that succeeded in overcoming intrinsic tumor suppressor mechanisms. If tumor elimination is only partially achieved, a state of equilibrium between malignant cells and the immune system ensues. Tumor cells can become dormant or accumulate mutations, while the immune system continues to exert selective pressure, thereby controlling tumor progress temporarily or eventually eliminating the cancer cells. If elimination does not occur, tumor cell variants resistant to the existent immune response eventually give rise to tumor progression, thereby initiating the escape phase and characterizing failure of tumor immune control. The contribution of NK cells in cancer immunoediting and clinical outcomes is now being appreciated (35).

Natural killer cells have proved to be critical for the eradication and inhibition of metastasis of cancer cells *in vivo* (36). Perforin protein (pfp) and/or IFN-γ knockout (KO) mice predominately develop B-cell lymphomas, especially after 1 year of age (older animals) with a combination of pfp and IFN-γ KO inducing an early onset of lymphoma, suggesting a synergistic immunosurveillance effect (37). Late age development of B-cell lymphoma and lung adenocarcinoma were also observed in TRAIL KO mice (38) and pfp KO mice (37), respectively. These findings support a role for NK-cell immunosurveillance of B-cell lymphomas as well as epithelial malignancies, through a combination of NK-cell-mediated cytotoxic activity, IFN-γ secretion, and TRAIL killing pathways. In humans, NK cells are particularly relevant in the prevention of tumor development. An 11-year follow-up of the general population for cancer incidence showed an association between reduced NK-cell cytotoxicity and increased risk of cancer (39). It has been postulated that NK cells are critical to the prevention of cancer development (elimination and equilibrium); however, once the tumor microenvironment is established (escape), suppressor factors such as TGF-β and IL-10 are induced and negative inhibitory receptors, such as the T-cell immunoglobulin-and-mucin-domain-containing molecule-3 receptor (TIM-3) on NK cells, that maintain an NK-cell anergic state (40) are upregulated. The induced aberrant expression of HLA-G (membrane-bound and soluble) and increased shedding of MICA (sMICA) seen in tumor cells (41, 42) can further suppress NK-cell antitumor immune responses. HLA-G interaction with ILT2 and CD94/NKG2A results in the inhibition of NK-cell cytotoxicity, IFN-γ secretion, and chemotaxis (43), while sMICA–NKG2D binding impairs NK-cell tumor-specific cytotoxicity, NKG2D expression, and homeostatic maintenance (42).

As a result of these immune deregulated events, NK-cell-associated suppressor factors are currently being considered as immunotherapeutic targets. TIM-3, for instance, is an immunoregulatory checkpoint expressed by most lymphocyte subtypes with critical and complex implications in cytotoxic NK cells (44, 45). Increased TIM-3 expression on NK cells has been shown as a marker of poorer prognosis in lung adenocarcinoma and other types of cancer and correlates with reduced NK-cell cytotoxicity. Blockade of TIM-3 is capable of restoring IFN-γ secretion and cytotoxicity of NK cells in lung cancer (46). Recent studies by Fowke et al. have shown that low expression of various inhibitory molecules on NK cells were associated with HIV viral control (47). Despite the complexity of the immune suppressive strategy of the tumor microenvironment, targeting these inhibitory checkpoint receptors shows potential to restore NK-cell functionality in the control or clearance of solid tumors (48). Currently, several trials are underway assessing the impact of Tim-3 blockade in cancer patients (http://ClinicalTrials.gov: NCT02817633; NCT02608268). However, such therapeutic tools are still in their infancy in the context of HIV-associated ADC or NADCs. Given the success of immunotherapy targeting the inhibitory receptors PD-1 and CTLA-4 against several malignancies (49), evaluation of the impact of NK-cell function following immune checkpoint blockade may have relevance in the setting of HIV and may serve a dual purpose in both HIV eradication and tumor clearance. Combining immunotherapy and NK-cell-based therapies is another potential targeted strategy and warrants further investigation in individuals with HIV-associated malignancies (50–53).

## NK-Cell Immune Control of HIV Infection during ART

HIV infection induces significant phenotypic changes and negatively impacts NK-cell cytotoxicity (54). Cytotoxic NK cells in aviremic HIV donors have impaired ADCC that is associated with a reduced expression of FcRIIIA, an activated phenotype represented by increased expression of CD38 and HLA-DR. Furthermore, NK cells are rarely found in lymph nodes, an important site of both HIV replication and B-cell transformation (55). Critical activating receptors important in cancer immunosurveillance such as NKp30 and NKp46 are downregulated (56) in HIV infection, and expansion of dysfunctional CD56− NK-cell subsets (57) persists even after cART.

Non-neutralizing anti-HIV-1 antibodies can mediate protection through ADCC in assays of HIV candidate vaccines in non-human primate models of HIV infection. Several studies have suggested that HIV-specific ADCC responses may be contributing to partial control of HIV viremia during acute infection. The early initial interest in the utility of NK-mediated ADCC *via* HIV-specific antibodies was to enhance the development of an HIV vaccine and novel therapies to suppress HIV replication. Interestingly, in the RV144 HIV vaccine Thai trial, the robust ADCC responses observed were associated with modest protective efficacy (58). It would be intriguing to determine the effects of NK-mediated ADCC in the recently launched HVTN 702 study that builds on the success of the RV144 trial in support of this correlate. In addition, recent studies suggest that cytokine stimulation can enhance direct NK cytotoxicity and NK-mediated ADCC of autologous HIV-infected CD4+ T cells (59–61). Therefore, modulating NK activity is a potential strategy to develop novel immunotherapies to prevent and possibly lead to the eradication of HIV.

## Influence of NK-Cell Immunity in HIV-Associated Malignancies

**Figure 1:**
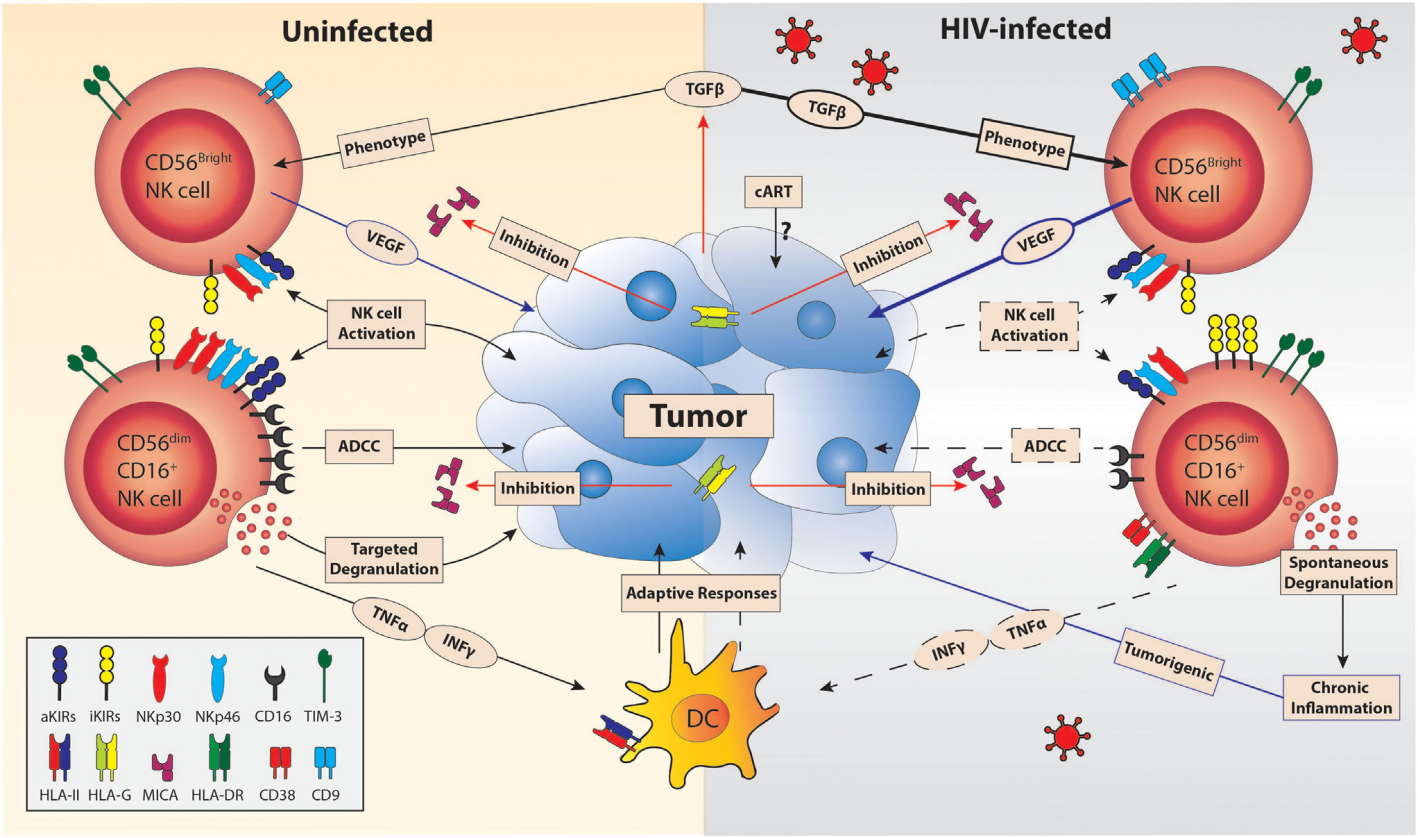
**Compounded effect of HIV infection on natural killer (NK) cell antitumor responses**. The tumor microenvironment constrains NK-cell functionality through the expression of tumor-derived transforming growth factor β (TGF-β) (68), shedding of MICA, and HLA-G (69). The limitations of antitumor mechanisms by NK cells are exacerbated in HIV infection. HIV infection reduces the surface expression of activation receptors (aKIRs, NKp30, and NKp46) and CD16 (56) while upregulating the expression of inhibitory receptors (iKIRs). The net result of the influence of HIV on NK-cell receptor expression further impairs NK-cell activation by cancer cell interaction and decreases tumor-directed antibody-dependent cell-mediated cytotoxicity (ADCC) responses (70). HIV infection decreases INF-γ and TNF-α production by NK cells despite HIV viral suppression by cART (71, 72), which will limit dendritic cell (DC) maturation and thus prevent efficient tumor-directed adaptive responses (73). The increased plasma TGF-β and loss of cell-specific degranulation of NK cells seen in HIV infection could lead to tumorigenicity *via* contributing to increased frequency of vascular endothelial growth factor (VEGF)-producing intratumoral NK cells (63) and occurrence of chronic inflammation, respectively (74). Furthermore, the affect of cART on tumor activity is yet to be explored. Blue lines represent responses that promote tumor growth, while responses that inhibit NK-cell function are indicated in red. Decreases are indicated by dashed lines and increases by bolded lines.

It is becoming apparent that NK cells may also contribute to tumorigenesis. The potential impact of such alterations in HIV malignancies is illustrated in Figure 1. Overexpression of activation markers on NK cells and spontaneous degranulation occurring during HIV infection may directly contribute to tumor development (33). Analysis of NK cells from patients with lymphoma demonstrated decreased levels of activating receptors in those with HIV compared to uninfected patients, suggesting that these cells might be less efficient to target cancer cells (62). NK cells can also present proangiogenic activity in the tumor microenvironment in a similar way to decidual NK (dNK) cells early on in pregnancy. In fact, in non-small cell lung cancer, the majority of tumor-infiltrating NK cells have a dNK-cell phenotype: CD56bright, CD9+, perforin low, and high production of vascular endothelial growth factor (VEGF) (63). Hypoxia and TGF-β secreted by tumor cells has a known immunomodulatory impact in the tumor microenvironment and induces VEGF secretion (64). *In vitro* exposure to TGF-β and hypoxia led to conversion of CD56dim NK cells into dNK-like cells (65). HIV infection leads to increased levels of TGF-β by monocytes (66) and T cells (67), suggesting that TGF-β may play a more prominent role in tumorigenesis during HIV infection.

The combination of (1) reduced expression of activating receptors and increased inhibitory receptors (e.g., TIM-3), (2) reduced ADCC, (3) reduced secretion of TNF-α and IFN-γ, and (4) development of pro-cancer features such as persistent activation, spontaneous degranulation, and production of VEGF suggests that NK cells may directly be associated with the increased cancer risk in the setting of ART-treated HIV infection. It is fair to speculate that targeted immunotherapies reversing specific NK cells deficits may be relevant for many HIV-related malignancies.

## NK-Cell-Based Immunotherapies Targeting HIV and HIV-Associated Malignancies

With the exception of the Berlin patient (75), and given the continued resurgence of virus in HIV-infected persons in various eradication approaches (76–78), it is clear that elimination of all latent HIV reservoirs is going to be critical to successfully achieve ART-free sustained HIV control or remission. Innovative approaches that are extrapolated from these studies and cases have lead to renewed interest in determining ways to bolster the host immune response and/or manipulate HIV target cells to render them refractory to infection. Since HIV and cancers have evolved sophisticated modalities to escape the host immune defense mechanisms, enhancing NK-cell function may serve as a promising tool as part of a multifaceted approach in the elimination of HIV as well as HIV-associated malignancies.

Recently, there has been renewed interest in harnessing HIV-specific ADCC responses as an HIV cure strategy. A monoclonal antibody (mAb) targeting the CD4-binding site on the HIV envelope spike (3BNC117) may have the potential to guide host immune effector cells to accelerate the clearance of HIV-1-infected cells by an FcyR-dependent mechanism (79, 80). In addition, Byrareddy et al. recently reported that a rhesusized mAb against α4β7 mediated sustained control of SIV infection in the absence of ART in non-human primates. This remarkable response was associated with increased cytokine-synthesizing NK cells (81). These studies, and others, highlight the potential of using mAbs through modulation of NK-cell-mediated activity as an exciting therapeutic tool to achieve sustained HIV remission and be beneficial in the context of HIV-associated tumors.

Natural killer-cell-based antitumor immunotherapeutic strategies targeting NK-cell activity have shown some promise in the oncology field (Table 1). Infusion of allogeneic or autologous NK cells has, to some degree, been successful in tumor clearance. Recently, single-chain variable fragment recombinant reagents, such as bispecific and trispecific killer cell engagers, are being considered as novel immunomodulators to enhance NK-cell function, antigen specificity, and *in vivo* expansion of these infused cells; however, these reagents need to be fully evaluated for clinical use (82–91). The efficiency of ADCC-mediated NK-cell responses is dependent on several factors from the mAbs themselves, NK-cell, and target cell status and also to the glycosylation state and the expression of glycosylation-specific ligands of both the NK-cell and target cells (92, 93). These features can be modified to enhance antiviral and immunomodulatory functions and/or the ability of the target cell to trigger or evade immunological recognition.

**Table 1:** **Therapeutic strategies utilizing natural killer (NK) cells in cancer immunotherapy that should be evaluated in the eradication of both HIV and HIV-associated malignancies**.

Strategy	Summary	Limitations	Reference
Allogeneic NK-cell-based immunotherapy	Freshly isolated or IL-2-stimulated NK-donor lymphocyte infusion	Requires further optimization to avoid graft-versus-host disease and to enhance efficiency	(94–103)

Autologous NK-cell-based immunotherapy	Activating endogenous NK cells and promoting their proliferation and function in patients using pro-inflammatory cytokine stimulation, or bispecific killer cell engagers (BiKEs) and trispecific killer cell engagers (TriKEs)	Low cytotoxic potential and possible side effects when using high doses of cytokines. BiKEs and TriKEs need to be fully evaluated for clinical use	(82–91, 104–106)

ADCC-based immunotherapy	Tumor-targeting monoclonal antibodies (e.g., anti-CD20, anti-HER-2, anti-GD2, anti-EGFR, and anti-GD2) or bispecific antibodies to induce antibody-dependent cell-mediated cytotoxicity (ADCC)	Requires tumor antigen-specific antibodies	(79, 107–111)

Immune checkpoint inhibitors-based immunotherapy	Blockade of NK-cell surface inhibitory receptors by specific antibodies (e.g., anti-PD-1, anti-NKG2A, anti-KIRs, anti-TIM-3, and anti-CTLA-4) in order to induce NK cells cytolytic activity	Possible side effects	(44, 112–118)

Genetically reprogrammed NK cells	Genetic modification of NK cells to induce the expression of activating receptors, silencing inhibitory receptors, inducing cytokine production, or genetic transferring of chimeric antigen receptors	Methods need further optimization	(119–141)

Several gene-editing technologies have been explored to genetically reprogram NK cells to optimize their persistence, expansion, migration, and cytotoxic capacity to improve the antitumor efficacy of primary human NK cells *in vivo*. With many challenges associated with most of these technologies, CRISPR/Cas9 nuclease system offers a new promising tool to gene-edit NK cells to improve their utility as a novel cell-based cancer immunotherapy strategy (142). Finally, latency reversal agents (LRAs) are being explored as a part of a “shock” approach to reverse cellular HIV latency and expose HIV reservoirs to immune-mediated clearance. Garrido et al. recently studied the impact of LRAs on the function of primary NK cells *ex vivo* and showed a heterogeneous mixed effect of different LRAs on antiviral activity, cytotoxicity, cytokine secretion, phenotype, and viability (143). Therefore, it will be important to comprehensively evaluate the impact of potential HIV curative strategies and elucidate the possible effect on NK cells against HIV-associated cancers.

## Conclusion

To achieve ART-free sustained HIV remission, it is likely that a multidisciplinary approach that includes a combination of immunotherapies and novel targeted approaches that will boost both the adaptive and innate immunity will be necessary. From this review, we posit that NK cells remain a very attractive therapeutic target to develop curative interventions for both HIV and HIV-associated malignancies.

## Author Contributions

LN and MA-M conceived and designed the review; LN, MA-M, and FL wrote the paper; and TP contributed to editing the review and designed and developed the figure.

## Conflict of Interest Statement

The authors declare that the research was conducted in the absence of any commercial or financial relationships that could be construed as a potential conflict of interest.
